# A new earless species of *Poyntonophrynus* (Anura, Bufonidae) from the Serra da Neve Inselberg, Namibe Province, Angola

**DOI:** 10.3897/zookeys.780.25859

**Published:** 2018-08-08

**Authors:** Luis M. P. Ceríaco, Mariana P. Marques, Suzana Bandeira, Ishan Agarwal, Edward L. Stanley, Aaron M. Bauer, Matthew P. Heinicke, David C. Blackburn

**Affiliations:** 1 Department of Natural Sciences, University of Michigan-Dearborn, 4901 Evergreen Road, Dearborn, Michigan 48128, USA University of Michigan-Dearborn Dearborn United States of America; 2 Department of Biology, Villanova University, 800 Lancaster Avenue, Villanova, Pennsylvania 19085-1699, USA Universidade de Lisboa Lisbon Portugal; 3 Museu Nacional de História Natural e da Ciência, Universidade de Lisboa, Rua da Escola Politécnica, 58, 1269-102 Lisboa, Portugal Villanova University Villanova United States of America; 4 Research Center in Biodiversity and Genetic Resources (CIBIO), InBIO, University of Porto, Rua Padre Armando Quintas 7, Vairão, 4485-661 Porto, Portugal University of Porto Porto Portugal; 5 Instituto Nacional da Biodiversidade e Áreas de Conservação (INBAC), Ministério do Ambiente de Angola, Centralidade do Kilamba, Rua 26 de Fevereiro, Quarteirão Nimi Ya Lukemi, edifício Q11, 3° andar, Angola Ministério do Ambiente de Angola Luanda Angola; 6 Department of Natural History, Florida Museum of Natural History, University of Florida, Gainesville, Florida 32611, USA University of Florida Gainsville United States of America

**Keywords:** Africa, Amphibia, columella, osteology, toad

## Abstract

African pygmy toads of the genus *Poyntonophrynus* are some of the least known species of African toads. The genus comprises ten recognized species endemic to sub-Saharan Africa, five of which are restricted to southwestern Africa. Recent field research in Angola provided new material for three species of *Poyntonophrynus*, including a morphologically distinctive population from the Serra da Neve Inselberg. Based on a combination of external morphology, high-resolution computed tomography scanning, and molecular phylogenetic analysis, the Serra da Neve population is described as new species that is nested within the genus. The most striking character that differentiates the newly described species from its congeners is the lack of a tympanic middle ear, a condition common in the family Bufonidae, but so far not known for *Poyntonophrynus*. The description of this new species from southwestern Angola reinforces the biogeographic importance of the region and further suggests that southwestern Africa is the cradle of diversity for this genus.

## Introduction

African pygmy toads of the genus *Poyntonophrynus* Frost et al., 2006, are a group of ‘true’ toads (family Bufonidae) that are endemic to sub-Saharan Africa (du Preez and Carruthers 2009, 2017). Similarities among what Poynton (1964) termed the *Bufo* ‘*vertebralis* group’ were recognized long before a genus was erected for them (Boulenger 1905, Mertens 1955, Poynton 1964, Tandy 1972, Poynton and Haacke 1993). Likewise, their similarity to those species today placed in the genus *Mertensophryne* has also long been acknowledged (Tandy 1972, Poynton 1996, Frost et al. 2006). However, the diversity and relationships of taxa in this group have remained contentious and confusing. Tandy (1972, Tandy and Keith 1972) revised Poynton’s (1964) ‘*vertebralis* group’ by excluding *Bufotaitanus* but adding both *B.lughensis* and *B.parkeri* due to similarities with *B.dombensis* and *B.fenoulheti*, respectively. The recognized diversity of this group has remained largely static since the description of *B.grandisonae* by Poynton and Haacke (1993) and the short summary by Poynton (1996).

Frost et al. (2006) recognized the genus *Poyntonophrynus* for a group of ten species that have typically been referred to as the ‘*vertebralis* group’ (Poynton 1964, Tandy 1972, Poynton and Broadley 1988, Poynton 1996). However, this was based solely on the results of Cunningham and Cherry (2004), who sampled three of these ten species finding that they form a clade exclusive of other African bufonids. Van Bocxlaer et al. (2010) first confirmed the long-suspected close phylogenetic relationship between the species recognized by Frost et al. (2006) as *Mertensophryne* and *Poyntonophrynus*. This result was expanded upon recently by Liedtke et al. (2017) who included new data for seven of the ten species included by Frost et al. (2006) in *Poyntonophrynus*.

**Figure 1.**
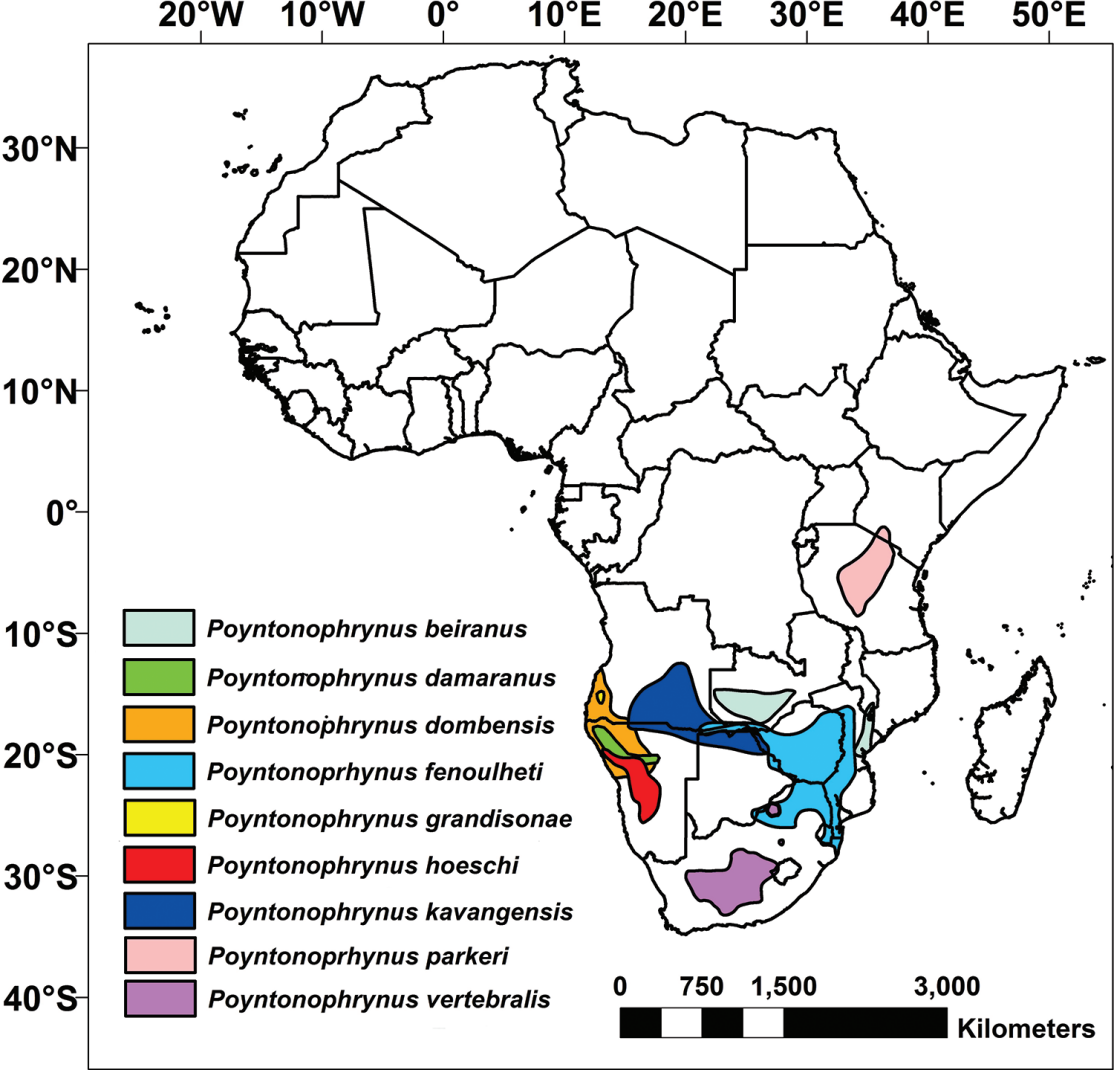
Known distribution of the nine currently accepted *Poyntonophrynus* species in Africa.

**Figure 2.**
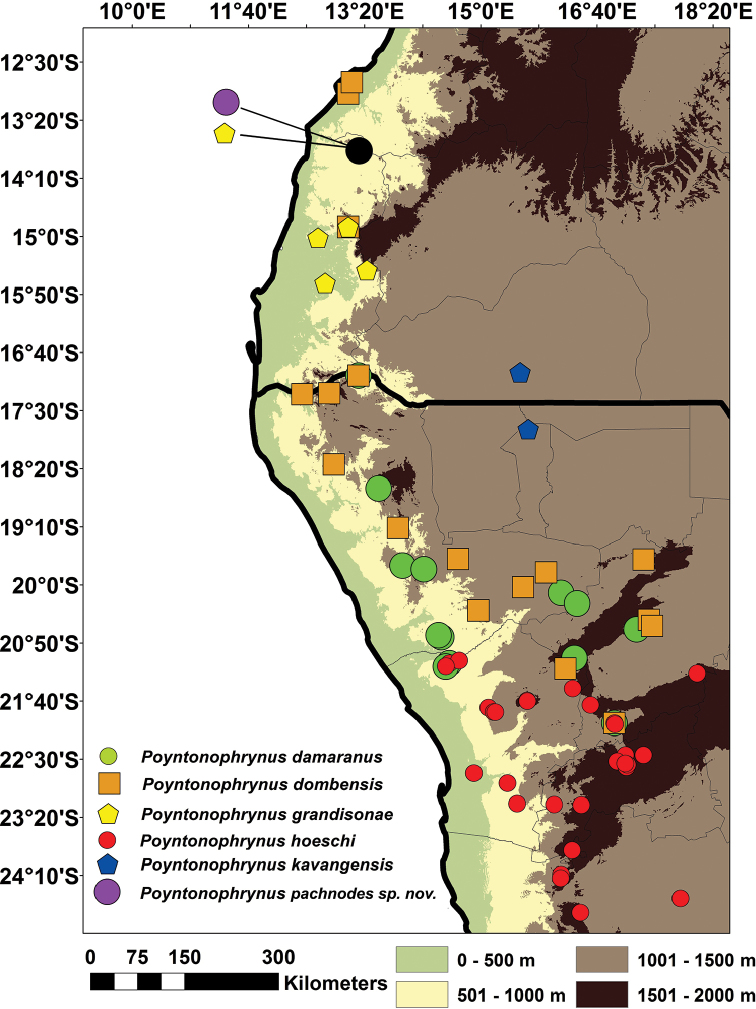
Bibliographic and confirmed museum records of the different *Poyntonophrynus* species occurring in southwestern Angola and northwestern Namibia.

There are currently ten recognized species in the genus *Poyntonophrynus*, all of which are small terrestrial toads with inconspicuous parotoid glands and lacking a tarsal fold. One East African species, however, *Poyntonophrynuslughensis* (Loveridge, 1932), was recently demonstrated to be more closely related to *Mertensophryne* (Liedtke et al. 2017). The remaining nine species are found in sub-Saharan Africa, with most of the diversity concentrated in Angola and Namibia. While the life history of the majority of these species is not well known, at least some species breed in shallow rocky pools and metamorphose relatively quickly (within ~3 weeks; Power 1926, Channing 1976, Channing and Vences 1999, Channing et al. 2012). The currently recognized species include the following: *P.beiranus* (Loveridge, 1932a), distributed in two populations, one in the coastal areas of Mozambique and another in southwestern and central Zambia; *P.damaranus* (Mertens, 1954), known from northwestern Namibia, but potentially also occurring in southwestern Angola; *P.dombensis* (Bocage, 1895), extending from southwestern Angola to central Namibia; *P.fenoulheti* (Hewitt & Methuen, 1912), with five described subspecies (*fenoulheti*, *obtusum*, *albiventris*, *rhodesianus* and *grindleyi*, all currently synonymized with the nominotypical form – see Frost 2017), occurring in western Mozambique, northeastern South Africa, Zimbabwe, the Caprivi Strip of Namibia, and portions of eastern and northern Botswana and southern Zambia; *P.grandisonae* (Poynton & Haacke, 1993), endemic to southwestern Angola (Namibe Province); *P.hoeschi* (Ahl, 1934), endemic to the central-western regions of Namibia; *P.kavangensis* (Poynton & Broadley, 1988), occurring in the Kavango River basin, in western Zimbabwe, northern Botswana, northern Namibia and southern Angola; *P.parkeri* (Loveridge, 1932b), restricted to northern Tanzania and southern Kenya; and *P.vertebralis* (Smith, 1848), the type species of the genus, occurring in central South Africa (Figure 1). Our current knowledge of the diversity of *Poyntonophrynus* remains limited and some species, such as *P.grandisonae* and *P.parkeri*, have not been documented since their original descriptions. Notably, five species of *Poyntonophrynus* occur in southwestern Africa, with four occurring in Namibia (*P.dombensis*, *P.hoeschi*, *P.damaranus* and *P.kavangensis*; Figure 2) and other four in southern Angola (*P.dombensis*, *P.fenoulheti*, *P.grandisonae*, and *P.kavangensis*). It is possible that other species of *Poyntonophrynus* also occur in Angola (Figure 2).

Despite being among the better explored regions in Angola, there are still new distributional records and undescribed species of reptiles and amphibians being discovered in the coastal lowlands of Namibe and Benguela provinces (Conradie et al. 2012, Ceríaco et al. 2016). These included the Slender Feather-tailed Gecko, *Kolekanosplumicaudus* (Haacke, 2008), the sole representative of the endemic genus *Kolekanos* Heinicke, Daza, Greenbaum, Jackman & Bauer, 2014; Haacke’s Sand Lizard, *Pedioplanishaackei* Conradie, Measey, Branch & Tolley, 2012, and Huntley’s Sand Lizard, *P.huntleyi* Conradie, Measey, Branch & Tolley, 2012; and the Kaokoveld Girdled Lizard, *Cordylusnamakuiyus* Stanley, Ceríaco, Bandeira, Valério, Bates & Branch, 2016. Namibe Province also harbors four other endemic lizard species (Ansorge’s Gecko, *Afrogeckoansorgii* (Boulenger, 1907), the Angolan Thick-Toed Gecko, *Pachydactylusangolensis* Loveridge, 1944, the Angolan Namib Day Gecko, *Rhoptropustaeniostictus* Laurent, 1964, and Bogert’s Dotted Blind Dart Skink, *Typhlacontiaspunctatissimusbogerti* Laurent, 1964) and the poorly known endemic Grandison’s Pygmy Toad, *Poyntonophrynusgrandisonae*. Some species known to occur in neighboring Namibia have also recently been found in Namibe Province, including the Large-Scaled Thick-Toed Gecko, *Pachydactylusscutatus* Hewitt, 1927, and the Damaraland Sand Frog, *Tomopternadamarensis* Dawood & Channing, 2002, previously known only from its type locality in Khorixas, Namibia (Ceríaco et al. 2016, Heinicke et al. 2017a).

During recent field research in Angola, we collected specimens representing several species of *Poyntonophrynus*. These include the second record for Angola of *P.dombensis*, the first specimens of *P.grandisonae* since the type series, and one undescribed species, with which this paper deals (Marques 2015). On Serra da Neve, an isolated inselberg in northern Namibe Province and the second highest peak in Angola (2489 m a.s.l. fide Pereira 1977), we collected specimens of a unique taxon of small toad. These specimens lack an externally visible ear, but otherwise are morphologically similar to species in the genus *Poyntonophrynus*. Despite considerable differences in size and shape, all described species of this genus have a visible tympanum, thus suggesting that the Serra da Neve population represents an unknown taxon. Based on our molecular phylogenetic and morphological studies, we describe this population from Serra da Neve as a new species of pygmy toad. We conclude by briefly discussing the biogeographic importance of the Serra da Neve Inselberg and the arid lowland areas of southwestern Angola.

## Materials and methods

### Specimens examined

Specimens collected for this study were preserved in 10% neutral buffered formalin in the field and transferred to 70% ethanol for storage. Liver tissue was removed before formalin fixation and preserved in either 95% ethanol or RNA Later. Specimens are deposited in the Florida Museum of Natural History at the University of Florida (**UF**; Gainesville, USA), the California Academy of Sciences (**CAS**; San Francisco, USA), the Instituto Nacional da Biodiversidade e Áreas de Conservação (**INBAC**; Kilamba-Kiaxi, Angola), and the Museu Nacional de História Natural e da Ciência (**MUHNAC**; Lisboa, Portugal). We made comparisons with specimens in the Museum of Comparative Zoology of Harvard University (**MCZ**; Cambridge, USA) as well as consulted data presented in the descriptions of *Poyntonophrynus* species and other relevant works (e.g., Poynton and Broadley 1988, Channing 2001, du Preez and Carruthers 2009, 2017).

### Molecular methods

Portions of the mitochondrial 16S gene and nuclear RAG-1 gene were sequenced for newly collected specimens of *Poyntonophrynusgrandisonae* and the undescribed samples from Serra da Neve. Methods of tissue extraction, PCR, and sequencing follow Heinicke et al. (2017b). DNA was extracted from preserved liver samples using the Qiagen DNeasy Blood and Tissue kit following the manufacturer’s protocol. PCR reactions were performed for 40 cycles at an annealing temperature of 50 °C, using the primer pair 16SA (5’-CGCCTGTTTATCAAAAACAT-3’) and 16SB (5’-CCGGTCTGAACTCAGATCACGT) (Palumbi et al. 1996) and the primer pair RAG1-C (5’-GGAGATGTTAGTGAGAARCAYGG) and RAG1-E (5’-TCCGCTGCATTTCCRATGTCRCA) (Biju and Bossuyt 2003). Following PCR, DNA was purified with Axygen AxyPrep magnetic beads, and purified DNA was sequenced at the University of Michigan DNA sequencing core.

Sequence assembly was performed using BioEdit 7.0.5.3 (Hall 1999), and new sequences were deposited in GenBank (Table 1). The new sequences were then integrated into a data set consisting of all *Poyntonophrynus*, *Mertensophryne*, and *Capensibufo* sequences used in Liedtke et al. (2017), which include 16S and RAG-1, but also sequences of 12S, CO1, ND2, and CXCR4 (Table 1). All previously sequenced species of *Poyntonophrynus* and *Mertensophryne* were included in that particular data set. Although Liedtke et al. (2017) provided the first genetic data for several species of *Poyntonophrynus*, there are no voucher specimens associated with these data to compare to our specimens (J. Streicher, pers. comm.). Sequences of each gene were aligned using ClustalX (Larkin et al. 2007). Following alignment, the best performing partition scheme was identified using PartitionFinder 2.1.1 (Lanfear et al. 2012), treating each gene and each codon position of the protein-coding genes as potentially unique character sets, and performing a greedy search on the 56 commonly used models of evolution.

A maximum likelihood phylogenetic analysis was performed using IQ-TREE 1.5 (Nguyen et al. 2015). Partition and model choice were based on the best-fitting scheme as determined by PartitionFinder, resulting in a scheme using the following 11 partitions and models: 12S/16S (GTR+I+G); CO1 position 1 (TrN+I+G); CO1 position 2/CXCR4 position 2 (TrN+I); CO1 position 3 (TIM+G); ND2 position 1 (GTR+G); ND2 position 2 (TIM+I); ND2 position 3 (GTR+G); RAG1 position 2/CXCR4 position1 (HKY); CXCR4 position 3 (HKY+G); RAG1 position 1 (TVM); RAG1 position 3 (TrN+G). Branch support was assessed with 1000 bootstrap replicates. The resulting tree was rooted using *Capensibufo*, which recent published phylogenies suggest to be closely related to but outside of a clade containing *Poyntonophrynus* and *Mertensophryne* (Liedtke et al. 2017).

**Table 1.** Sample identifications and GenBank accession numbers of sequences used in phylogenetic analyses. Specimen ID acronyms: KTH – Krystal Tolley Field collection; HF – Harith Farooq field collection; MTSN – Museo Tridentino di Scienze Naturali, Trento, Italy; BM – Natural History Museum, London, United Kingdom; MCZ – Museum of Comparative Zoology, Harvard University, USA; JM – John Measey field collecion; AACRG – Louis Du Preez field collection; AMB – Aaron M. Bauer field collection; BP – Anonymous field collection; VG – Miloslav Jirků field collection; UF – University of Florida, Museum of Natural History, Gainesville, USA.

Species	Specimen ID	12S	16S	CO1	ND2	CXCR4	RAG1
* Capensibufo rosei *	KTH 09-335	KF664868	KF665294	KF665706	–	KF665976	KF666159
* Mertensophryne anotis *	HF 33	KY555630	KY555643	KY555662	–	–	KY555712
* Mertensophryne howelli *	MTSN-T2202	KF664964	KF665247	KF665531	–	KF666045	KF666383
* Mertensophryne lindneri *	BM 2002.394	KF664736	KF665426	KF665790	–	KF665953	KF666333
* Mertensophryne loveridgei *	MCZ A-32084	KF664924	KF665338	KF665572	KY555685	KF665947	KF666463
* Mertensophryne micranotis *	MCZ A-32087	KF665020	KF665240	KF665579	KY555703	KF666123	KF666378
* Mertensophryne nyikae *	MCZ A-137123	KY555631	KY555647	KY555657	–	–	KY555722
* Mertensophryne taitana *	JM 773	KF664809	KF665047	KF665612	KY555705	KF665995	KF666310
* Mertensophryne usambarae *	MTSN 9541	KF665026	KF665336	KF665800	–	KF666115	KF666360
* Mertensophryne uzunguensis *	BM 2002.157	KF664717	KF665170	KF665699	–	FJ882720	KF666366
* Poyntonophrynus beiranus *	HF 30	KY555625	KY555650	KY555665	–	–	KY555721
* Poyntonophrynus damaranus *	n/a	–	AF220905	–	AF463793	–	–
* Poyntonophrynus dombensis *	n/a	AF220857	AF220907	–	AF463794	–	–
* Poyntonophrynus fenoulheti *	AACRG 1598	KF664732	KF665265	KF665592	KY555710	KF666066	KF666249
* Poyntonophrynus grandisonae *	AMB 10337	–	MH469716	–	–	–	MH469717
* Poyntonophrynus hoeschi *	n/a	AF220828		–	–	–	–
* Poyntonophrynus kavangensis *	BP-001	KY555627	KY555648	KY555658	–	–	–
* Poyntonophrynus lughensis *	VG001	KY555626	KY555641	KY555659	KY555700	KY555666	KY555723
*Poyntonophrynuspachnodes* sp. n.	UF 184184	–	MH469718	–	–	–	MH469719

### Morphological methods

Osteological data were obtained from six specimens of the Serra da Neve population, 18 additional *Poyntonophrynus* specimens, and five specimens of *Mertensophryne* (see Suppl. material 1). We performed high-resolution x-ray computed tomography (CT-scanning) at the University of Florida’s Nanoscale Research Facility. We used a Phoenix v|tome|x M (GE Measurement & Control, Boston, USA) scanner with a 180 kv x-ray tube with a diamond-tungsten target and with the following settings: Voltage = 75–100 kV, Current = 200 mA, 0.2–0.3 second detector capture time, averaging three images per rotation, and voxel resolution of 17–31μm (see Suppl. material 1: Table 1 for details). Raw 2D x-ray data were processed using the datos|x software v. 2.3 with post-processing, analyses (including segmentation), and visualization conducted using VG StudioMax v. 3.1 (Volume Graphics, Heidelberg, Germany). Both tomogram stacks (in TIFF format) and surface models for the CT-scanned specimens are available in MorphoSource (see Suppl. material 1).

Measurements and external morphological data followed the standardized protocols presented by Watters et al. (2016). Measurements were taken with an electronic caliper accurate to 0.01 mm, rounded to 0.1 mm. The following measurements were taken of adults:

**SVL** snout-vent length;

**HW** head width, taken at the angle of the jaw;

**HL** head length, from the posterior of the jaws to the tip of the snout;

**ED** eye diameter, horizontally from the anterior to posterior corner of the eye;

**IND** internarial distance, shortest distance between the inner margins of the nostrils;

**EN** eye-nostril distance, from the anterior corner of the eye to the posterior margin of the nostril;

**TD** tympanum diameter, greatest horizontal width of the tympanum;

**SL** snout length, distance from the tip of the snout to the anterior corner of the eye;

**NS** snout-nostril length, distance from the center of the external nares to the tip of the snout;

**IOD** interorbital distance, the shortest distance between the anterior corners of the orbits;

**UEW** upper eyelid width, greatest width of the upper eyelid margins, measured perpendicular to the anterior-posterior axis;

**FLL** forearm length, from the flexed elbow to the base of the outer palmar tubercle;

**HAL** hand length, from the base of the outer palmar tubercle to the tip of finger IV;

**Fin4L** finger IV length, from the proximal edge of the palmar tubercle to the tip of finger IV;

**TL** tibiofibula length, distance from the outer surface of the flexed knee to the heel/tibiotarsal inflection;

**THL** thigh length, distance from the vent to the knee;

**FL** foot length, from the base of the inner metatarsal tubercle to the tip of toe IV;

**Toe4L** Toe IV length, from the metatarsal tubercle to the tip of toe IV.

## Results

**Figure 3.**
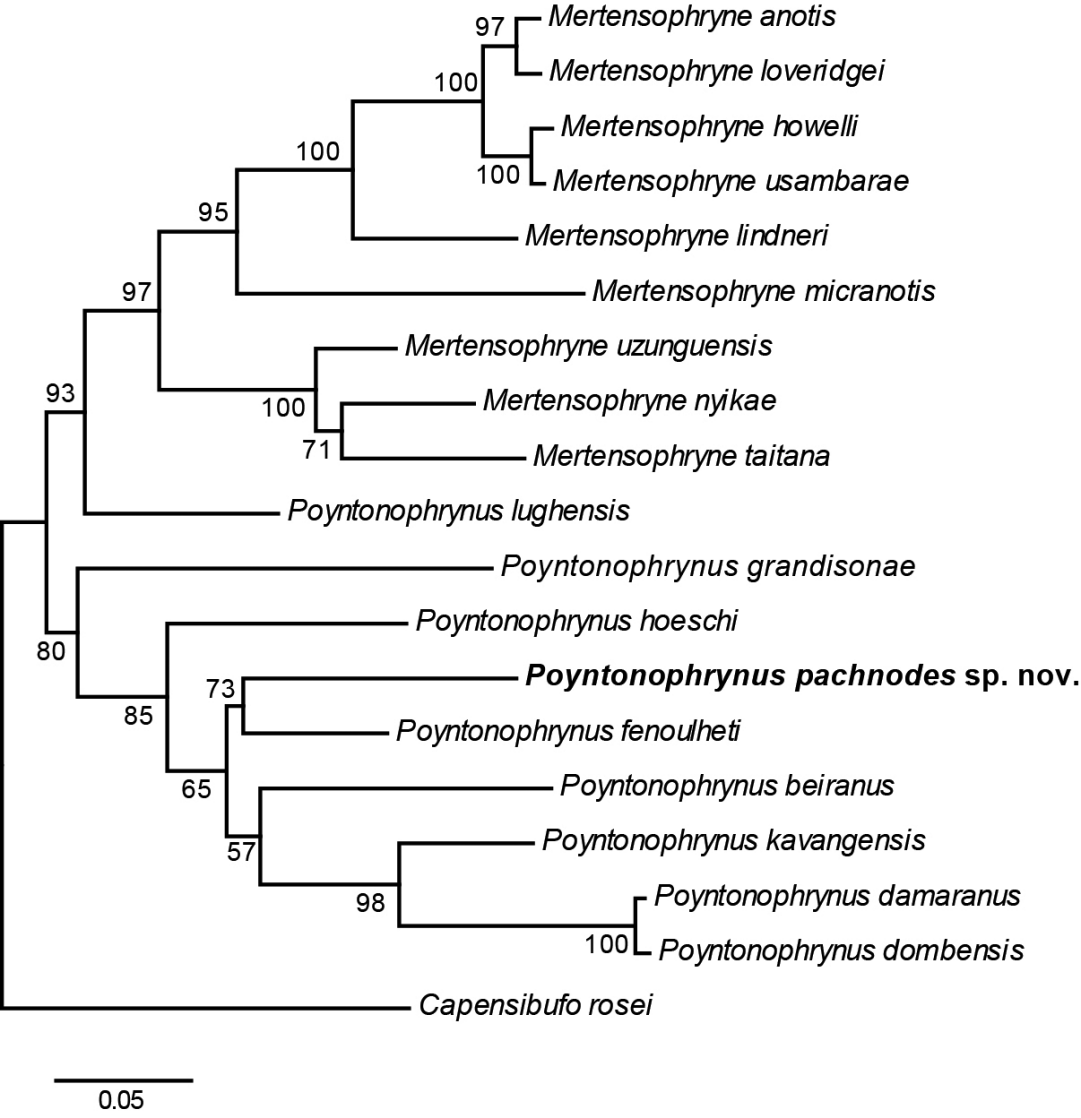
Maximum likelihood tree of the genus *Poyntonophrynus*. Support values at nodes are bootstrap percentages; scale bar in substitutions per site.

Molecular phylogenetic and morphological analyses suggest that the pygmy toads collected from the Serra da Neve represent an undescribed species of *Poyntonophrynus*. The phylogenetic analysis reveals that the Serra da Neve population is nested within the genus *Poyntonophrynus* and is most closely related to *P.fenoulheti* (Figure 3) with weak bootstrap support (73%). As expected, since we used data from Liedtke et al. (2017), the eastern African species *P.lughensis* is more closely related to *Mertensophryne* than to its putative congeners. Within the well-supported clade (bootstrap support 80%) of other species of *Poyntonophrynus*, *P.grandisonae* is sister to remaining congeners. However, as our data do not include overlapping sequences between *P.grandisonae* and *P.hoeschi*, our result should be taken with caution.

Morphologically, the Serra da Neve taxon is typical of *Poyntonophrynus* by being a small toad, lacking a tarsal fold, and having indistinct, flattened parotoid glands. However, this population also exhibits distinct morphological characters that clearly distinguish it from all described species of the genus, most notably the lack of tympanum and a combination of several morphological and coloration characters (Table 2). Based on these differences, this population is described here as a new species.

**Table 2.** Comparison between the *Poyntonophrynuspachnodes* sp. n. with its congeners. Data based on our observations of freshly collected material, original descriptions, and the revisionary works of Poynton and Broadley (1988), Channing (2001), and du Preez and Carruthers (2009, 2017).

	*P.pachnodes* sp. n	* P. dombensis *	* P. hoeschi *	* P. damaranus *	* P. vertebralis *	* P. kavangensis *	* P. beiranus *	* P. fenoulheti *	* P. parkeri *	* P. grandisonae *
**Maximum Snout-vent length**	31 mm	40 mm	37 mm	37 mm	36 mm	33 mm	28 mm	43 mm	30 mm	46 mm
**Parotoid glands**	Inconspicuous, flattened	Conspicuous, flattened	Conspicuous to completely flattened	Conspicuous, with well defined edges but flattened	Inconspicuous, flattened	Inconspicuous, of constant width, with outer edge straight and not extending below pupil	Inconspicuous to hardly discernible	Inconspicuous, flattened	Inconspicuous to hardly discernible	Inconspicuous to hardly discernible
**Tympanum**	Not present	Present, conspicuous, diameter smaller than internarial distance	Present, inconspicuous	Present, inconspicuous	Present, inconspicuous	Present, conspicuous, diameter smaller than internarial distance	Present, inconspicuous	Present, conspicuous, diameter smaller than internarial distance	Present, inconspicuous	Present, conspicuous, diameter equal or bigger than internarial distance
**Skin on snout**	Granular	Smooth	Smooth	Granular	Granular	Smooth	Smooth	Granular	Granular	Smooth
**Skin on venter**	Granular	Smooth to slightly granular	Smooth to slightly granular	Granular	Slightly granular	Granular	Very granular	Smooth to slightly granular	Smooth to slightly granular	Smooth to slightly granular
**Vertebral Line**	Not present	Usually not present	Not present	Not present	Present	Present	Usually present	Usually not present	Not present	Not present
**Dorsal coloration**	Dark brown with coppery to brown mottling and dark brown blotches especially in anterior regions , and a pale scapular patch	Light to dark brown, with small dark blotches and pale scapular patch	Brown to reddish-brown with light and dark marking	Olive-brown with symmetrical to irregular dark blotches, dark interorbital band	Grey to brown with orange and reddish markings, pale scapular patch, single or a pair of patches in lower back	Three pairs of dark patches with dark interorbital band, light colored scapular patch with pale projections upper eyelids	Dark grey and with a pale scapular patch	Light grey to brown, with small scattered dark blotches, pale scapular patch, sometimes with a single or a pair of patches in lower back	Light grey to brown, with small scattered dark blotches, pale scapular patch, sometimes with a single or a pair of patches in lower back	Light-grey to brown, with dark patches in the scapular and poster parts of the dorsum, top of the head cream
**Ventral coloration**	Cream-colored, juveniles whitish with distinct black blotches	Immaculate	Immaculate	Yellowish-white	Whitish with distinct black blotches	Cream-colored	Pale without marking	Immaculate, occasionally with black blotches or spots	Cream-colored	Immaculate
**Webbing**	Toes without a margin of web, webbing between toes vestigial	Scanty, only reaching base of fourth toe	Toes with distinct margin of webbing	Moderately well developed, three to three and a half phalanges of longest toe free of webbing	Toes scantly webbed, two segments of the third toe are free of web	Toes scantly webbed with serrated margins, broad web between toes three and four	Two or three segments of the longest toe free of webbing	Scantly webbed	Toes without a margin of web, webbing between toes vestigial	Toes without a margin of web, webbing between toes vestigial
**Subarticular tubercles**	Double	Usually double	Double	Well defined, usually double	Usually double	Usually double	Usually double	Usually double	Single	Single, except distal tubercle of third finger
**Metatarsal tubercles**	Inner three times bigger than outer	Smaller than outer but substantially larger than other palmer tubercles.	Inner similar in size or smaller than outer	Inner bigger than the outer	Inner three times bigger than outer	Inner elongated towards the medial surface below base of first finger. Outer , rounded to triangular shape. Inner two to three times smaller than the outer	Inner not present, but a tubercle at base of first finger may be slightly larger than other tubercles. Outer markedly enlarged	Inner three times bigger than outer	Inner two to three times smaller than outer	Inner bigger than the outer

### Poyntonophrynus pachnodes sp. n.

Taxon classificationAnimaliaAnuraBufonidae

http://zoobank.org/3899209A-5389-4CE4-A806-92D5CE61ACF7

**Figure 4.**
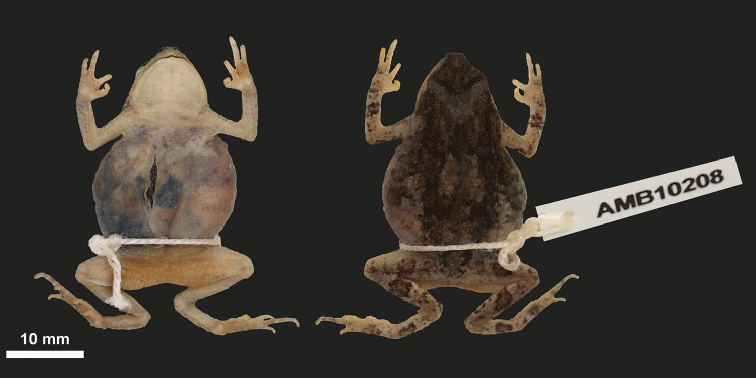
Ventral and dorsal view of the holotype (UF 184184) of *Poyntonophrynuspachnodes* sp. n.

**Figure 5.**
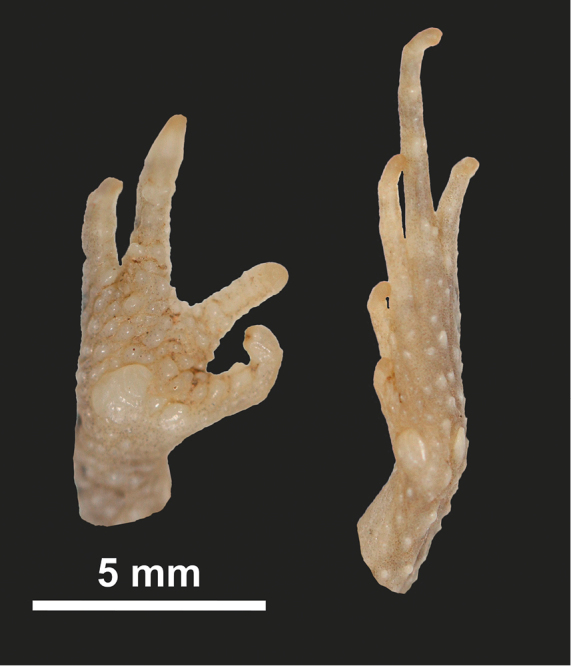
Ventral view of the right hand and left foot of the holotype (UF 184184) of *Poyntonophrynuspachnodes* sp. n.

**Figure 6.**
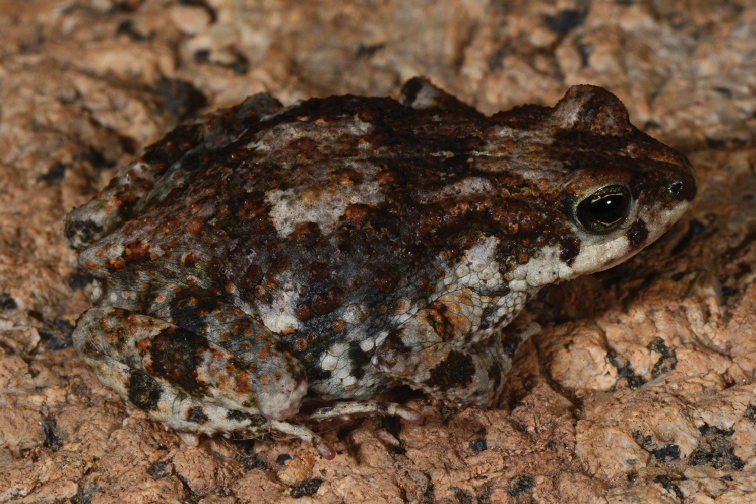
Photograph in life of *Poyntonophrynuspachnodes* sp. n., paratype UF 184183 (AMB 10191). Photo by Ishan Agarwal.

**Figure 7.**
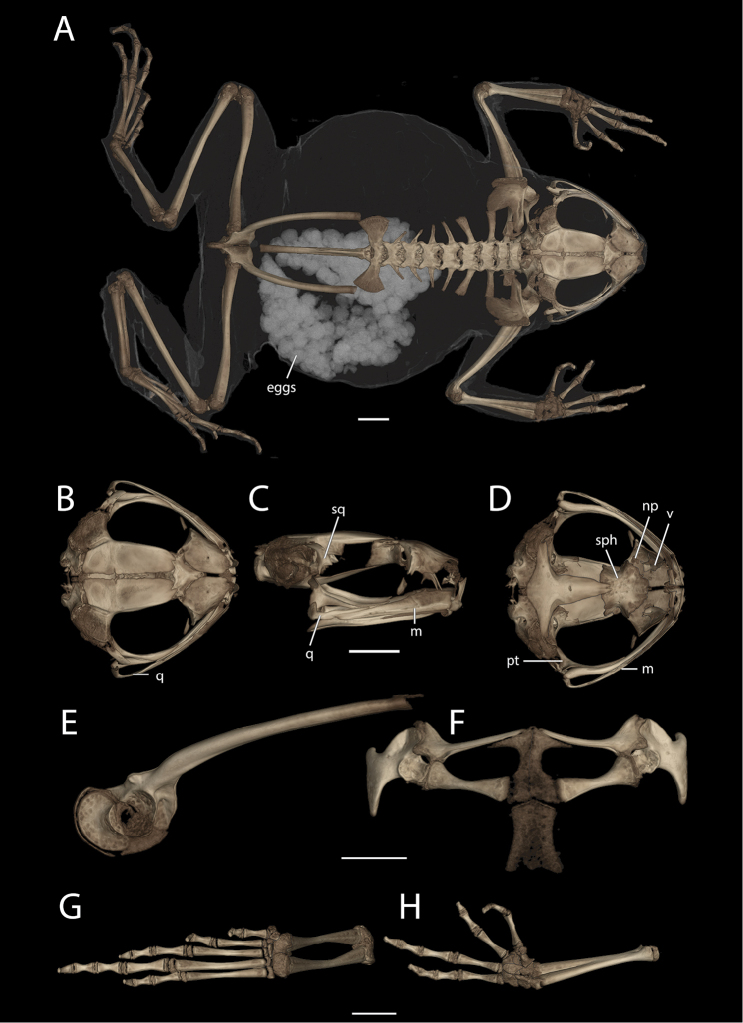
High-resolution Computed Tomography reconstructions showing the skeleton of the holotype (UF 184184) of *Poyntonophrynuspachnodes* sp. n. in dorsal view (**A**), skull in dorsal (**B**), right lateral (**C**) and ventral (**D**) views, pelvis in right lateral view (**E**), pectoral girdle in ventral view (**F**), and right foot (**G**) and right hand (**H**) in ventral views. Selected skeletal elements are labeled based on discussion in the text. Panel A also highlights the eggs that are visible within the oviducts. Abbreviations: m – maxilla; np – neopalatine; pt – pterygoid; q – quadrate; sph – sphenethmoid; sq – squamosal; v – vomer. Scale bars in each row equal 2 mm.

Figs 4 , 5 , 6 , 7 , Table 3

#### Holotype.

A female, UF 184184 (field number AMB 10208), collected on Serra da Neve (-13.77704 S, 13.25905 E; datum WGS 84; 1488 m a.s.l.), 18 November 2016, by Luis M. P. Ceríaco, Suzana Bandeira and Ishan Agarwal.

#### Paratypes.

A male UF 184183 (field number AMB 10191), a juvenile CAS 262729 (field number AMB 10207), a female CAS 262730 (field number AMB 10210), all with the same collecting data as the holotype, a male INBAC/AMB 10209, and male MUHNAC/MB04-000999 (field number AMB 10219) with same locality and collector data as the remaining type series but collected 19 November 2016.

#### Diagnosis.

*Poyntonophrynuspachnodes* sp. n. is a small-bodied bufonid that lacks tarsal folds, a character that distinguishes it from bufonids in Angola except *Mertensophryne* and *Poyntonophrynus*. It differs from all *Mertensophryne* in having inconspicuous parotoid glands, compared to pronounced parotoid glands that form a shelf in the scapular region of *Mertensophryne*, and in lacking reduction of the phalanges (Grandison, 1981). The newly described species differs from all other members of the genus *Poyntonophrynus* in lacking a tympanum and columella.

#### Description of the holotype.

Small (SVL 31.4 mm), robust, stout and gravid female, with moderately robust limbs (Figure 4–5; All measurements in Table 3); head triangular, wider than long; snout projecting slightly beyond upper jaw; rostral tip straight in dorsal, ventral and lateral views; eyes projecting laterally just beyond eyelids and approximately flush with margins of head in dorsal view; eye projecting about 50% above dorsal margin of head in lateral view; interorbital distance 1.1 times eye diameter; pupil large and ellipsoid in life and preservative; loreal region concave; naris small, triangular, directed dorsolaterally; canthus rostralis short, eye diameter 1.2 times eye-narial distance; eye diameter 1.6 times naris to rostral tip; internarial region flat with rounded lateral margins; interorbital distance approximately 1.7 times internarial distance; tympanum and middle ear structures (tympanum, columella) absent. Marginal and vomerine teeth absent.

**Table 3.** Measurements of the type series of *P.pachnodes* sp. n. For measurement abbreviations see Materials and methods section. All measurements in mm.

**Catalog number**	**UF 184184**	**UF 184183**	**CAS 262730**	**CAS 262729**	**MUHNAC/MB04-000999**	**INBAC/AMB 10209**
Type status	Holotype	Paratype	Paratype	Paratype	Paratype	Paratype
SEX	Female	Male	Juvenile	Female	Male	Male
HW	9.9	9.5	8.9	9.2	9.9	10.3
SVL	31.4	31.3	29.7	28	33.7	30.0
TL	11.7	12.1	10.9	11.6	12.3	12.5
IOD	3.2	3.2	3.3	3.3	4.5	4.2
HL	8.7	8.1	8.8	9.1	11.7	10.6
ED	2.8	2.9	2.7	2.9	3.0	3.0
IND	1.9	2.3	2.2	1.9	2.5	2.0
EN	2.6	2.2	2.4	2.6	2.8	2.7
FL	11.8	12.0	11.7	11.7	12.1	12.5
THL	11.6	11.0	10.7	11.2	12.0	13.0
SL	3.9	3.7	4.2	4.5	3.9	4.0
HAL	7.4	7.1	6.8	6.9	7.4	7.2
FLL	7.2	6.0	6.5	6.6	6.7	7.8
UEW	2.8	2.2	2.6	2.9	2.2	2.4
FIN4L	4.6	4.9	4.0	4.5	4.7	4.3
TOEL4L	5.9	7.3	6.2	6.7	7.7	6.1
NS	1.7	1.8	1.0	1.2	1.5	1.4

Skin of venter with evenly scattered miniscule asperities; skin of gular region smooth; skin of limbs, dorsal and dorsolateral surface of head and body with scattered tubercles, being larger on dorsum; inconspicuous and flattened parotoid glands, elliptical, and weakly elevated, placed dorsolaterally and extending from posterior corner of mouth to level of axilla.

Limbs and digits well-developed; digits of both manus and pes stout; tarsal fold not present; relative length of fingers: III > I > IV = II; finger tips not expanded, but with rounded tips; fingers with rounded, prominent double subarticular tubercles; two palmar tubercles distinct and widely separated from one another, one at ventromedial surface of first finger and other at proximal plantar surface, latter being about 4 times larger than first; webbing between manual digits absent; relative length of toes IV > III > II > I = V; toe tips slightly expanded; toes with prominent, single, and subarticular tubercles; webbing between toes vestigial, not reaching first joint of first phalanx; prominent and globular inner metatarsal tubercle, length 50% of first toe length; tarsal tubercle prominent and projecting, near medial edge and positioned at distal fourth of tarsus.

#### Coloration.

In life, dorsal ground color dark brown with coppery to brown mottling and dark brown blotches especially in anterior regions (Figure 6); whitish chevron extending between eyes (scapular patch), directed posteriorly (faded but visible in preservative); iris dark green with dark brown pupil (dark grey and pale gray in preservative, respectively); snout similar in color and pattern to dorsum; dorsal surface of forelimbs and fingers III and IV whitish with dark brown blotches; dorsal surface of fingers I and II white; posterior to head, distinctive white blotch at midbody; dorsum and lateral surface dark brown speckled with coppery markings, becoming faint towards venter; dorsal surface of hind limbs (thighs and crus) greyish white with distinctive dark brown markings; three dark blotches on thigh, crus and feet touching when legs flexed; base color of dorsal foot grayish, with scattered dark brown blotches, extending across toes; region surrounding cloaca cream colored. Lateralmost margin of upper jaw white interrupted by brown markings posterior and anterior to eye; throat immaculate white; ventral surface of forearm and arm whitish; ventral surface of hand and fingers white; venter unpigmented and whitish; anterior part of thighs and ventral crus cream colored; ventral side of legs and plantar surface of pes unpigmented and whitish in appearance.

#### Variation.

The coloration of the remaining paratypes do not differ in any important details from those of the holotype. Measurements of the remaining type series in Table 3.

#### Osteology.

Based on CT scan of UF 184184 (holotype; gravid female; Figure 7). The skull is wider than long, incompletely ossified, and lacks ornamentation on the dermal roofing bones. The jaw joint is anterior to the otic region. The parotic plate is incompletely ossified but synostosed to the frontoparietal. The premaxillae lack teeth, and have both a robust pars dentalis and a robust alary process that is taller than wide and widely separated from the nasals. The maxillae lack teeth and are weakly concave (with apex directed labially). The quadratojugals are thin and elongate with a broad articulation with the maxillae and reaching anterior to the articulation of the maxilla and pterygoid. The pterygoids are slender, with a long medially curved anterior ramus with a broad articulation with the adjacent maxilla, a short posterior ramus approaching, but not articulating with, the cartilages of the jaw joint, and a short medial ramus approaching but not articulating with the prootic. Vomers are large and plate-like, lacking teeth. The neopalatine is a thin flat rod, not articulating with adjacent bones. The curved and triradiate septomaxillae are present at the anterior margin of the nasal capsule. The prominent sphenethmoid is coosified across the midline and visible in dorsal view between the nasals and frontoparietals. The parasphenoid narrows anteriorly and exhibits a small bifurcation at its rostral extent. The squamosals are greatly reduced with only the dorsalmost otic region and a small reduced zygomatic ramus present. The prootic is poorly ossified and has a poorly defined fenestra ovalis. Neither a bony operculum nor a columella is present. The posteromedial processes of the hyoid are ossified and slender, and expanded weakly at their articulation with the cartilaginous hyoid plate.

There are eight distinct, procoelous, non-imbricating presacral vertebrae that are not synostosed. The atlas lacks transverse processes and has widely separated cotyles. The sacrum is procoelous with laterally expanded transverse processes. No sesamoid is observed at the sacroiliac joint. The urostyle is long and thin with a weakly developed dorsal ridge.

The pectoral girdle is fermisternal with widely spaced and slender coracoids. The clavicles are slender, nearly reaching one another. The scapulae are stout, directed laterally but strongly curving dorsally at their lateral extent. Neither an ossified sternum nor omosternum is present.

The pelvic girdle comprises the ilium, pubis, and ischium, which are not synostosed to one another. The acetabulum is incompletely ossified. The shaft of the ilium is long and slender, lacking a dorsal crest, and with a small prominent dorsal protuberance. The synostosed ischia form a broad posteriorly directed plate.

The radioulna is shorter than the humerus. The radiale and ulnare are large and subequal in size, though the other carpals are difficult to distinguish as they are incompletely ossified. The phalangeal formula for the manus is 2–2–3–3, and there is both a single ossified prepollex and a small palmar sesamoid. The tips of the terminal manual phalanges are weakly expanded into small knobs. The tibiofibula is slightly shorter than the femur. There are two small distal tarsals. The phalangeal formula for the pes is 2–2–3–4–3 and there is a single small ossified prehallux and a plantar sesamoid. The tips of the terminal pedal phalanges are weakly expanded as in the fingers.

#### Distribution and ecology.

**Figure 8.**
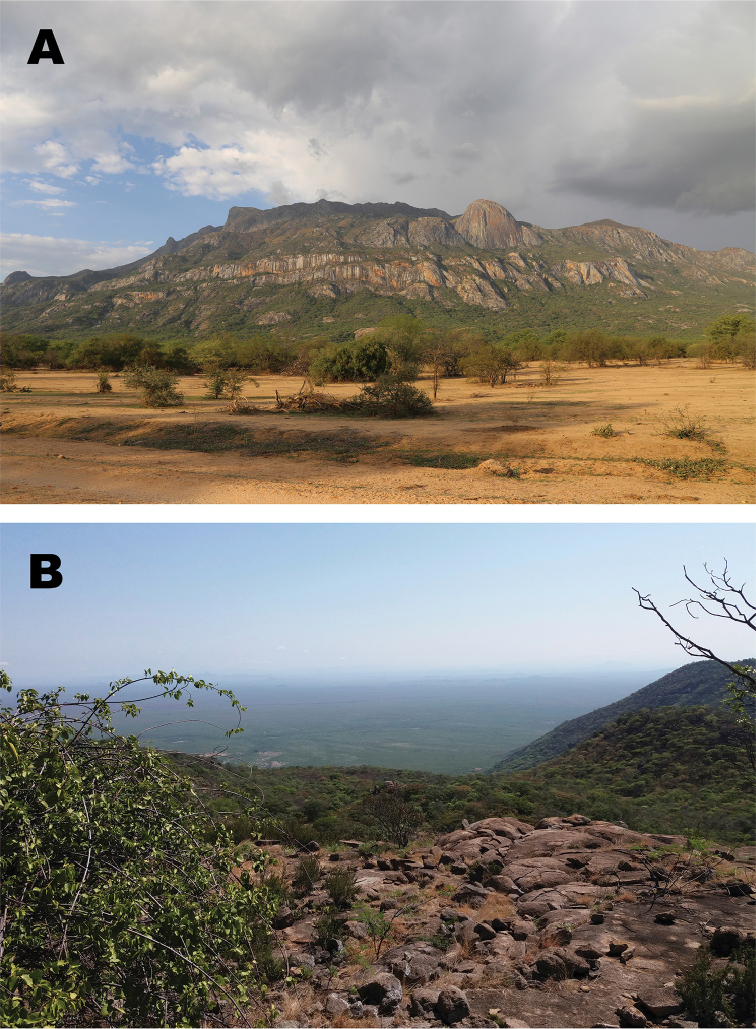
Southern view of the base of the Serra da Neve Inselberg (**A**), and habitat of the type locality at about 1500 m a.s.l. (**B**). Photos by Luis Ceríaco.

The species is currently only known from the Serra da Neve Inselberg (Figs 2, 8) in northern Namibe Province. Specimens were found on moist soil under rocks and leaf-litter at dusk in a semi-open miombo forest area. Grandvaux-Barbosa (1970) considered that Serra da Neve is characterized by a “sparse Miombo,” dominated by trees of the genera *Julbernardia* spp. and *Brachystegia* spp., and shrubs like *Combretum* spp. or *Annona* spp., which were observed at the site. The species was found sympatrically with the frogs *Sclerophrysgutturalis* and *Tomopternatuberculosa*, the lizards *Agamaschacki*, *Trachylepissulcata*, *Chondrodactyluspulitzerae*, *Hemidactylusbenguellensis*, *Pachydactylusangolensis*, and *Helioboluslugubris*, and the snake *Hemirhagerrhisviperina*.

#### Etymology.

The specific name *pachnodes* (Gr.) means “frosty” and is used as an adjective (Brown 1954). This is a reference to both the cool climate at the higher elevation where this species occurs (1488 m) and that Serra da Neve (Port.) translates to “mountain of snow.” We suggest “Serra da Neve Pygmy Toad” and “Sapo Pigmeu da Serra da Neve” as the English and Portuguese common names respectively.

## Discussion

Despite several decades of interest in African pygmy toads (e.g., Poyton 1964, Tandy 1972, Poynton and Haacke 1993), there has been little attention paid to the systematics of the species now referred to as *Poyntonophrynus*. Until recently, there has been no serious attempt to evaluate the monophyly of this genus. The molecular phylogenetic analysis of Liedtke et al. (2017) demonstrates that *P.lughensis* is more closely allied to *Mertensophryne*, and if true then this species should be treated as part of that genus. However, as the sample used in that analysis is not associated with a voucher specimen to confirm the identification, we refrain from taxonomic changes. It still remains uncertain whether *B.parkeri*, which Tandy (1972, Tandy and Keith 1972) included in the ‘*vertebralis* group,’ should be included in *Poyntonophrynus*; Tandy (1972) provided no specific arguments regarding this assignment. While we have contributed new molecular genetic data for *P.grandisonae*, we still lack phylogenetic information for several species. In lieu of new genetic resources for molecular phylogenetic analyses, a thorough systematic revision based on morphological data, including osteology, is needed to evaluate the monophyly of *Poyntonophrynus* and the relationships among its species.

**Figure 9.**
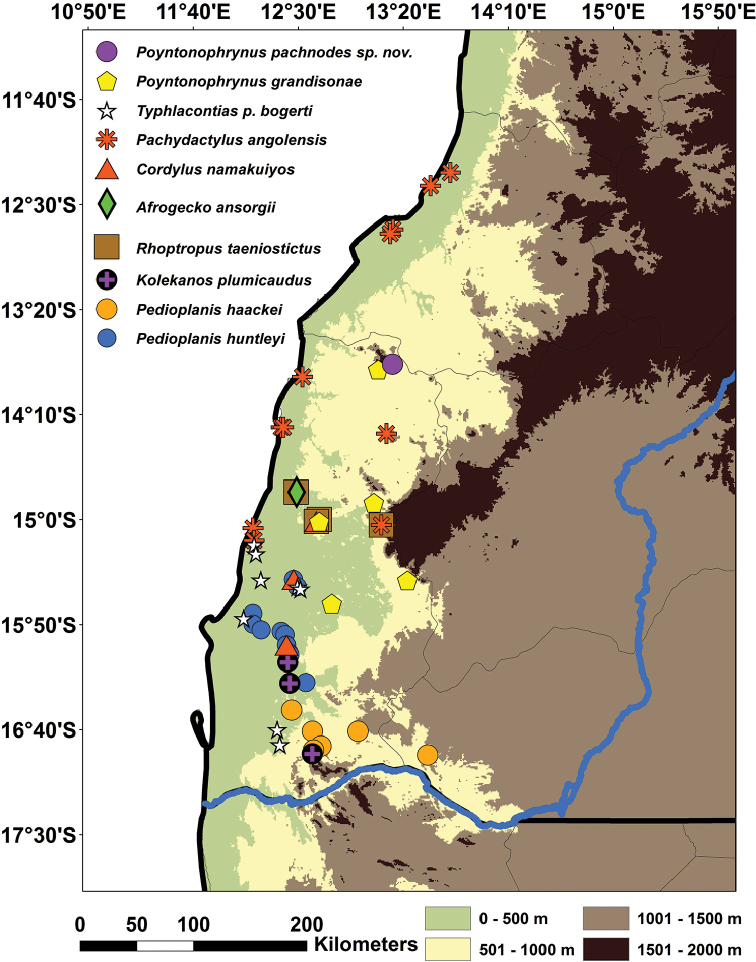
Bibliographic (Marques 2015), museum and new field records of the amphibian and reptile taxa endemic to the coastal arid lowlands of southwestern Angola. The blue line represents the Cunene/Kunene River.

Unlike most African anuran taxa, the species diversity of *Poyntonophrynus* is concentrated in arid southwestern Africa. Five species (*P.damaranus*, *P.dombensis*, *P.grandisonae*, *P.hoeschi*, and *P.pachnodes* sp. n.) are strictly endemic to this region, whereas the others (*P.vertebralis*, *P.beiranus*, *P.kavangensis*, *P.fenoulheti*, and *P.parkeri*) are found in arid or mesic habitats extending across southern and eastern Africa. This pattern of species distributions, combined with the fact that *P.grandisonae* represents the earliest known diverging lineage within the genus, suggests that the origin of this group might have been in southwestern Africa. While a highly unusual pattern for an anuran, this is similar to several squamate taxa for which the arid zones of southwestern Angolan and northwestern Namibia form a center of endemism and diversity (Roll et al. 2017, Marques 2015). Southwestern Angola hosts a large number of endemic species of lizards, including one endemic genus, and ongoing work reveals additional cryptic and endemic diversity among several lizard taxa (Figure 9).

In addition to *Poyntonophrynus*, the few examples of amphibians found in these xeric areas include *Phrynomantisannectens*, several species of *Sclerophrys*, and *Tomopterna* (Ceríaco et al. 2016, Heinicke et al. 2017a). Within Namibe Province, there are 91 documented species of reptiles, representing one-third of the reptile species known for Angola, but only 15 amphibian species, which represent <13% of the amphibian diversity of the country (Marques 2015). Neighboring Benguela Province has similar species diversity (102 reptiles, 36 amphibians) and contains similar habitats. The herpetofaunal diversity of these provinces mostly comprises taxa found in southern Africa (Marques 2015). Though Namibe Province is generally well explored, little is known of the diversity of its inselbergs. The biodiversity of Serra da Neve remains largely undocumented with only two expeditions to it in the last few years (Barker et al. 2015, this study), according to available literature. Other inselbergs and highlands found along the Angolan escarpment, including Mount Moco (Huambo Province) and the highlands of Gabela-Seles and the Kumbira Forest (Kwanza Sul Province), are known to harbor a significant number of endemics and relict populations of various taxa (Marx 1956, Hall 1960, Hall and Moreau 1970, Huntley 1974, Sekercioglu and Riley 2005, Maiato 2009, Mills 2010, Mills et al. 2011, Cáceres et al. 2015, Carleton et al. 2015, Gonçalves and Goyder 2016, Svensson et al. 2017). While the biogeographic affinities of these areas are not completely understood, there is a general pattern of relictual distributions for taxa otherwise found in central or eastern Africa. Yet due to its southern geographic position, surrounding arid habitats, and the limited existing data on occurring taxa, it appears that the affinities of the flora and fauna of Serra da Neve have more similarities to taxa found typically in southern Africa, as for example representatives of the gekkonid genera *Rhoptropus*, *Chondrodactylus* and *Pachydactylus*, or southwestern African endemics including *Hemirhagerrhisviperina* and *Trachylepislaevis*; this new species of *Poyntonophrynus* provides an additional example. We are, however, still far from understanding the biogeographic affinities of Serra da Neve.

**Figure 10.**
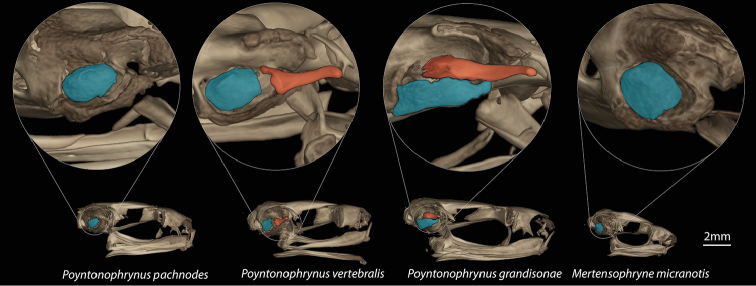
Comparisons of the skulls of *Poyntonophrynuspachnodes* sp. n. (UF 184184) in comparison to *P.vertebralis* (MCZ A-10007), *P.grandisonae* (AMB 10340), and *Mertensophrynemicranotis* (CAS 162553). Light blue indicates the otic plate and orange indicates the columella, which is absent in both *P.pachnodes* and *Mertensophryne*.

The osteology of both *Poyntonophrynus* and its sister taxon *Mertensophryne* are poorly documented in comparison to other African bufonid taxa. The skeleton of *P.pachnodes* exhibits features characterized by Grandison (1981) as common in the *vertebralis* group, though she was not explicit about the species she included in that group. Those features include the reduction or loss of the shaft of the squamosal and an elongate quadratojugal that attains the articulation between the maxilla and the pterygoid at its anterior extent (Poynton 1991, Graybeal and Cannatella 1995). Similar to the condition documented by Grandison (1981) for *P.vertebralis*, the neopalatines are reduced in *P.pachnodes* (Figure 7D), in contrast to its well-developed state in *Mertensophryne* (Grandison 1981, Poynton 1991). All of the species that are currently recognized as *Mertensophryne* lack external ears (Tandy 1972, Tandy and Keith 1972, Frost et al. 2006), though it is not clear whether all of these species might also lack other structures such as the columella (Tihen 1960). *Poyntonophrynuspachnodes* does not exhibit reduction in the number of presacral vertebrae, a character which is present at least some *Mertensophryne* (e.g. Figure 10; Tihen 1960, Grandison 1978), nor does it exhibit any of the ornamentation found in the dorsal skull bones, as in *Mertensophryneanotis* (Poynton 1991). The newly described species represents the first known case of complete loss of the Tympanic Middle Ear (TME) within the genus *Poyntonophrynus* (Figure 10), though all of the species in the sister-genus *Mertensophryne* lack the TME (Tandy and Keith 1972). This condition is generally rare among amniotes, but it is fairly common among anurans, including the Bufonidae (Pereyra et al. 2016). In some cases, the TME is not completely lost, and some portions of the middle ear may remain. The reduction of TME structures in anurans follows a consistent pattern (Pereyra et al. 2016): the absence of more medial structures (e.g., columella) is associated with the absence of lateral structures such as the tympanic annulus and membrane, but the absence of the lateral structures are not necessarily associated with the reduction or loss of the columella. The evolutionary loss of TME and the alternative auditory mechanisms that might be used by these taxa have received substantial recent attention (Boistel et al. 2011, 2013, Thomas et al. 2014, Pereyra et al. 2016, Goutte et al. 2017, Womack et al. 2017). Given the loss of the TME in *Mertensophryne* and at least once species of *Poyntonophrynus*, this clade may provide an opportunity for further examination of the causes of loss and reduction of the TME and other correlated features (e.g., Womack et al. 2018).

## Supplementary Material

XML Treatment for Poyntonophrynus pachnodes

## Supplementary materials

Supplementary material 1 CT-Scan settings Data type: specimens data Explanation note: CT-Scan settings for the different specimens used in this study. This is an open access article distributed under the terms of the Creative Commons Attribution License (CC BY 4.0), which permits unrestricted use, distribution, and reproduction in any medium, provided the original author and source are credited.Luis M. P. Ceríaco, Mariana P. Marques, Suzana Bandeira, Ishan Agarwal, Edward L. Stanley, Aaron M. Bauer, Matthew P. Heinicke, David C. Blackburn
