# Supplementary material for: A new earless species of Poyntonophrynus (Anura, Bufonidae) from the Serra da Neve Inselberg, Namibe Province, Angola
Source: Zookeys. 2018 Aug 8;(780):109–36. doi: 10.3897/zookeys.780.25859 (PMC6093966; doi:10.3897/zookeys.780.25859)
Supplement: Supplementary material 1 — CT-Scan settings [file zookeys-780-109-s001.docx]

| **Species** | **Accession number** | **Voxel size (μm)** | **Voltage (kV)** | **Current (mA)** | **Detector caputure time (S)** | **DOI** |
| --- | --- | --- | --- | --- | --- | --- |
| *Mertensophryne lonnbergidi* | UF 92078 | 25,02667 | 100 | 200 | 0,2 | [doi:10.17602/M2/M48867](https://doi.org/10.17602/M2/M48867) |
| *Mertensophryne lonnbergidi* | UF 92079 | 25,02667 | 100 | 200 | 0,2 | [doi:10.17602/M2/M48551](https://doi.org/10.17602/M2/M48551) |
| *Mertensophryne micranotis* | CAS 162553 | 23,00351 | 100 | 200 | 0,333 | [doi:10.17602/M2/M25590](https://doi.org/10.17602/M2/M25590) |
| *Mertensophryne taitana* | UF 92081 | 29,07923 | 100 | 200 | 0,2 | [doi:10.17602/M2/M48868](https://doi.org/10.17602/M2/M48868) |
| *Mertensophryne taitana* | UF 92083 | 29,07923 | 100 | 200 | 0,2 | [doi:10.17602/M2/M48869](https://doi.org/10.17602/M2/M48869) |
| *Poyntonophrynus beiramus* | BMNH 1947-2-21-10 | 17,25247 | 75 | 200 | 0,2 | [doi:10.17602/M2/M43501](https://doi.org/10.17602/M2/M43501) |
| *Poyntonophrynus dombensis* | UF 184864 | 29,48882 | 90 | 150 | 0,2 | doi:10.17602/M2/M50747 |
| *Poyntonophrynus dombensis* | MCZ-A-148576 | 28,75639 | 100 | 200 | 0,2 | [doi:10.17602/M2/M43489](https://doi.org/10.17602/M2/M43489) |
| *Poyntonophrynus dombensis* | MCZ-A-148575 | 28,75639 | 100 | 200 | 0,2 | [doi:10.17602/M2/M43490](https://doi.org/10.17602/M2/M43490) |
| *Poyntonophrynus dombensis* | MCZ-A-22395 | 31,14352 | 100 | 200 | 0,2 | [doi:10.17602/M2/M43492](https://doi.org/10.17602/M2/M43492) |
| *Poyntonophrynus dombensis* | MCZ-A-22397 | 31,14352 | 100 | 200 | 0,2 | [doi:10.17602/M2/M43491](https://doi.org/10.17602/M2/M43491) |
| *Poyntonophrynus fenoulheti* | MCZ-A-137800 | 26,47706 | 80 | 150 | 0,2 | doi:10.17602/M2/M43545 |
| *Poyntonophrynus fenoulheti* | MCZ-A-35986 | 24,6571 | 100 | 200 | 0,2 | [doi:10.17602/M2/M43537](https://doi.org/10.17602/M2/M43537) |
| *Poyntonophrynus grandisonae* | UF 184185 | 25,52291 | 80 | 200 | 0,2 | [doi:10.17602/M2/M43462](https://doi.org/10.17602/M2/M43462) |
| *Poyntonophrynus grandisonae* | CAS 262731 | 25,52291 | 80 | 200 | 0,2 | [doi:10.17602/M2/M43454](https://doi.org/10.17602/M2/M43454) |
| *Poyntonophrynus grandisonae* | CAS 262732 | 27,58316 | 80 | 200 | 0,2 | [doi:10.17602/M2/M43463](https://doi.org/10.17602/M2/M43463) |
| *Poyntonophrynus lughensis* | MCZ-A-87529 | 26,74951 | 100 | 200 | 0,2 | [doi:10.17602/M2/M43455](https://doi.org/10.17602/M2/M43455) |
| *Poyntonophrynus parkeri* | MCZ-A-16335 | 26,20143 | 100 | 200 | 0,2 | doi:10.17602/M2/M50706 |
| *Poyntonophrynus parkeri* | MCZ-A-16336 | 28,31356 | 100 | 200 | 0,2 | [doi:10.17602/M2/M43456](https://doi.org/10.17602/M2/M43456) |
| *Poyntonophrynus pachnodes* sp. nov. | UF 184183 | 24,38813 | 80 | 200 | 0,2 | [doi:10.17602/M2/M43457](https://doi.org/10.17602/M2/M43457) |
| *Poyntonophrynus pachnodes* sp. nov. | CAS 262729 | 21,54254 | 80 | 200 | 0,2 | [doi:10.17602/M2/M43458](https://doi.org/10.17602/M2/M43458) |
| *Poyntonophrynus pachnodes* sp. nov. | UF184184 | 23,14957 | 80 | 200 | 0,2 | [doi:10.17602/M2/M43460](https://doi.org/10.17602/M2/M43460) |
| *Poyntonophrynus pachnodes* sp. nov. | CAS 262730 | 20,30233 | 80 | 200 | 0,2 | [doi:10.17602/M2/M43461](https://doi.org/10.17602/M2/M43461) |
| *Poyntonophrynus pachnodes* sp. nov. | UF 184186 | 22,4262 | 80 | 200 | 0,2 | [doi:10.17602/M2/M43494](https://doi.org/10.17602/M2/M43494) |
| *Poyntonophrynus pachnodes* sp. nov. | UF 184187 | 31,88616 | 80 | 200 | 0,2 | [doi:10.17602/M2/M43469](https://doi.org/10.17602/M2/M43469) |
| *Poyntonophrynus vertebralis* | MCZ 10918 | 20,27859 | 100 | 200 | 0,2 | [doi:10.17602/M2/M43470](https://doi.org/10.17602/M2/M43470) |
| *Poyntonophrynus vertebralis* | MCZ-A-100007 | 27,63829 | 100 | 200 | 0,2 | [doi:10.17602/M2/M43471](https://doi.org/10.17602/M2/M43471) |
| *Poyntonophrynus vertebralis* | MCZ-A-100008 | 27,63829 | 100 | 200 | 0,2 | [doi:10.17602/M2/M43472](https://doi.org/10.17602/M2/M43472) |
